# DREMECELS: A Curated Database for Base Excision and Mismatch Repair Mechanisms Associated Human Malignancies

**DOI:** 10.1371/journal.pone.0157031

**Published:** 2016-06-08

**Authors:** Ankita Shukla, Ahmed Moussa, Tiratha Raj Singh

**Affiliations:** 1 Department of Biotechnology and Bioinformatics, Jaypee University of Information Technology (JUIT), Waknaghat, Solan, H.P., 173234, India; 2 LabTIC Laboratory, ENSA-Tangier, Abdelmalek Essaadi University, BP1818 Route Ziaten, 90 000, Tangier, Morocco; University of Lausanne, SWITZERLAND

## Abstract

DNA repair mechanisms act as a warrior combating various damaging processes that ensue critical malignancies. DREMECELS was designed considering the malignancies with frequent alterations in DNA repair pathways, that is, colorectal and endometrial cancers, associated with Lynch syndrome (also known as HNPCC). Since lynch syndrome carries high risk (~40–60%) for both cancers, therefore we decided to cover all three diseases in this portal. Although a large population is presently affected by these malignancies, many resources are available for various cancer types but no database archives information on the genes specifically for only these cancers and disorders. The database contains 156 genes and two repair mechanisms, base excision repair (BER) and mismatch repair (MMR). Other parameters include some of the regulatory processes that have roles in these disease progressions due to incompetent repair mechanisms, specifically BER and MMR. However, our unique database mainly provides qualitative and quantitative information on these cancer types along with methylation, drug sensitivity, miRNAs, copy number variation (CNV) and somatic mutations data. This database would serve the scientific community by providing integrated information on these disease types, thus sustaining diagnostic and therapeutic processes. This repository would serve as an excellent accompaniment for researchers and biomedical professionals and facilitate in understanding such critical diseases. DREMECELS is publicly available at http://www.bioinfoindia.org/dremecels.

## 1. Introduction

DNA repair mechanisms are regulatory bioprocesses through which a cell) identifies and corrects damages in the DNA, which is necessary for maintaining genome integrity. Depending on the type of damage, various DNA repair strategies, such as base excision repair (BER), nucleotide excision repair, mismatch repair (MMR), non-homologous end joining (NHEJ), and homologous recombination repair (HRR), have evolved to restore the lost information [1]. Defective repair mechanisms can lead to the accumulation of genetic or epigenetic mutations, thereby increasing the risk of various diseases and disorders. Studies have revealed that defective MMR and BER mechanisms are involved in many cancers, while their major role has been reported in two disease covered in this study [2–4].

The primary disorder associated with these repair mechanisms (specifically MMR) is an autosomal dominant genetic condition called Lynch syndrome (LS), also known as hereditary nonpolyposis colorectal cancer (HNPCC) [5]. LS occurs through germline mutation in MMR genes and is associated with tumors exhibiting microsatellite instability (MSI). LS is a major cause of many cancers, predominantly colorectal cancer (CRC), while some reports for its association with endometrial cancer (EC) have also been published [6–7]. For a patient with LS, the risk of CRC is 52%–82% (mean age 44–61 years) and that of EC is 25%–60% (mean age 48–62 years) [7]. Strafford JC showed in their study that tumors containing MMR gene mutations associated with LS often have a characteristic histologic appearance exhibiting microsatellite instability (MSI), which depict DNA mismatch repair gene dysfunctioning [8]. Numerous studies including expression arrays [9], miRNA arrays [10], comparative genome hybridisation arrays [11], chip-on-chip [12], methylation arrays, mutation analysis, genome-wide association studies, proteomics analysis, integrated functional genomics analysis and related bioinformatics, and biostatistical analysis have been conducted for the genes that get altered in these cancers and in LS [13]. These in-vitro and in-silico techniques provide bulk data regarding cancer-related genes, which facilitate researchers in deciphering the mechanisms underlying disease progression.

Although ample biomedical literature provides evidence for various genes involved in CRC, EC, and LS; but to our knowledge, there is no such resource that provides information specifically about these malignancies. In view of it, there was a need for such a resource which provides all the above mentioned information at one stop. Therefore, we developed a unique portal named DREMECELS (DNA REpair MEchanism in Colorectal, Endometrium and Lynch Syndrome) containing vital information related to these diseases. DREMECELS is a manually curated database of genes involved in repair mechanisms associated with the diseases. The database comprises of diversified data, such as basic gene and protein information, functional annotation, literature references, associated transcription factors, conserved domains information, gene ontology (GO) information, miRNA information, interacting partners, pathways, methylation, drug details, copy number variation (CNV), somatic mutations, and provides suitable links to various primary external resources. Moreover, we present a multiple sequence alignment (MSA) analysis that could help find conserved and variable regions in sequences, which can be used to identify functionally important sites. Furthermore, BLAST is integrated to assist users in aligning query sequences against sets of genes available in DREMECELS. DREMECELS provides information on gene markers associated with diseases, which could be of high relevance to researchers working on DNA repair-linked diseases and disorders, and particularly on BER and MMR mechanisms involved in CRC, EC, and LS.

## 2. Materials and Methods

Data concerning cancer-causing genes were collected manually from literature and various standard resources. Molecular and genetic events, such as methylation, drug sensitivity, gene interaction studies, and gene ontologies, have been captured and are pivotal to the database collection. After collecting the non-redundant set of genes, other information, such as respective proteins, transcription factors, pathways, annotations, conserved domains, disease type, literature references, structure IDs, GO, drug details, methylation data, CNV, somatic mutations, and miRNAs was procured and integrated into the dataset. After data collection, the database was designed considering the features of the collected data.

### 2.1 Database Design

The database design has 12 non-redundant tables: the gene data table, which contains detailed information on all non-redundant set of genes where gene ID is the primary key; the disease table, which contains information on the concerned disease; the conserved domains table, which contains information on the functional units of the proteins; the GO table, which contains the gene annotation information; the miRNA table, which contains miRNA information; the pathways table, which contains information on the pathways associated with the disease; the PubMed table, which stores literature references; the transcription factor table, which contains information on gene regulatory factors; the methylation table, which contains epigenetic mechanism data (hypo and hypermethylation); the drug sensitivity data table, which contains information on all the available drugs; the CNV table, which contains information on copy number alterations related to the gene; and the somatic mutations table, which contains information on polymorphisms related to the gene.

### 2.2 Resources used for data collection

The data relating to the proteins and their sequences for disease-associated genes was collected from UniProt [14]. Close homologues of the sequences were found, and MSA was performed using MUSCLE, a program for performing MSA with decreased time and space complexity [15]. The conserved domain database is used for locating conserved regions/domains of a protein sequence that functions independently [16]. Gene annotation was performed using Gorilla and WebGestalt tools, which are used for functional enrichment analysis, information retrieval, organisation, visualisation, and statistical analysis of large sets of genes [17,18]. miRNAs, which are involved in post-transcriptional regulation of gene expression, were collected from miRBase [19]. Biological pathways are crucial for regulating gene expression; therefore, we have externally linked KEGG pathways to DREMECELS to find the regulatory mechanism for every set of genes [20]. The literature was extensively searched using the PubMed database, and related references and appropriate links were included. Transcription factors were taken from GeneCards [21] also integrated as they are crucial for regulating the transcription rate.

**Fig 1:**
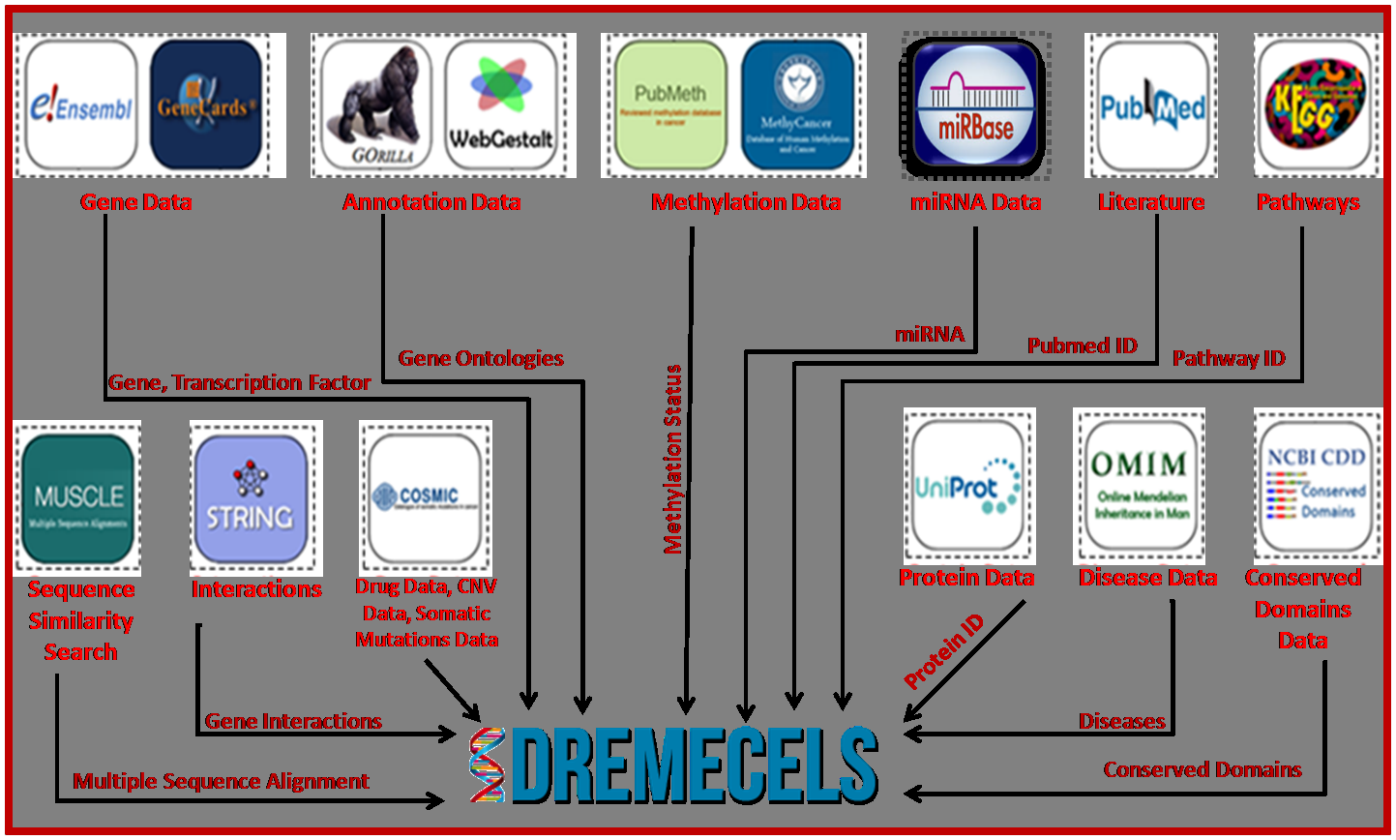
Back-end data collection resources. Gene data has been collected through ENSEMBL and GeneCards, protein data from UniProt, disease data from OMIM, conserved domains from NCBI CDD, pathways from KEGG, interaction data from STRING, gene ontologies from GORILLA and WebGestalt, miRNA data from miRBase, drug data, cnv data and somatic mutations data from COSMIC, methylation data from PubMeth and MethyCancer, similarity search has been performed through MUSCLE and relevant references from PubMed.

Methylation is the most studied epigenetic modification and thus is included in the database as an important feature. It regulates gene expression, and therefore can be proved a crucial biomarker for cancer detection and a therapeutic intervention target. Aberrant DNA methylation patterns, such as hypo and hypermethylation, are widely studied because of their involvement in various human malignancies. Therefore, we compiled a methylation pattern for CRC, EC, and LS from the methyl cancer database, PubMeth, and previous studies, and the most relevant data were incorporated in the database [22,23] (Table 1). Drug sensitivity occurs because of natural variations in individual’s drug metabolism. For drug sensitivity, data were collected for 140 drugs targeting CRC, EC, and LS from the catalogue of somatic mutations in cancer (COSMIC) [24]. CNV and somatic mutations data were also collected from COSMIC covering the acquired (somatic) alterations in tumor DNA [24]. CNV which are structural variations, are evident as a result of duplication or deletion via germ line or somatic event [25,26], while in somatic mutations single base-pair in genome differs between members of a species [27]. The overall process of extensive data collections for various parameters and features through diverse sources are shown in Fig 1.

**Table 1:** No. of genes implicated in different levels of Methylation.

Methylation status	Disease	No. of genes	Name of genes
**Methylated**	Colorectal cancer	32	TP53, MGMT, MPG, APC, MLH1, PMS1, EXO1, ATM, PTGS2, BCL2, KRAS, CDX2, TERT, FHIT, RB1, HNF1A, WRN, SMAD2, DCC, DKK1, IGF2, STK11, BAX, WNT5A, RASSF2, TWIST1, AXIN1, ERBB2, MYO1A, MEIS1, SFRP4, HLTF
	Endometrial cancer	9	MGMT, BRCA1, MLH1, MSH6, PMS1, RB1, CTNNB1, ABCB1, RPS6KA6
	Lynch syndrome (HNPCC)	3	BRCA1, MLH1, PMS1
**Hypomethylated**	Colorectal cancer	2	IGF2, MUC5AC
**Hypermethylated**	Colorectal cancer	11	MSH2, TDG, OGG1, PTEN, ERCC1, XPC, WRN, IGF2, ARID1A, IDO1, SDC1
	Endometrial cancer	2	PTEN, IGF2
	Lynch syndrome (HNPCC)	1	MSH2

### 2.3 Database Development

After successful designing, the database was developed by integrating the data in MYSQL, a relational database management system that is used as backend, and the web interface was built using HTML, JavaScript, CSS, and PHP. It is anticipated that the accrued information is expected to be critical for both researchers and clinicians in understanding and determining the molecular mechanisms underlying these diseases.

### 2.4 Biological and Molecular behavior studies

Furthermore, we classified all cancer-related genes according to the molecular function of each protein and the biological process in which it is involved. It was inferred from the molecular functions that most genes have transcription regulatory, tumor suppressor, DNA binding, enzymatic activities, and cell cycle control, thereby suggesting the significance of the gene products in cancer pathways. Regarding biological processes, gene products are involved in metabolic processes, response to stimulus, signaling, multicellular organismal process, and developmental processes. Therefore, information related to biological processes may be useful for understanding the cell behavior and its transition to the diseased state.

## 3. Results and Discussions

**Fig 2:**
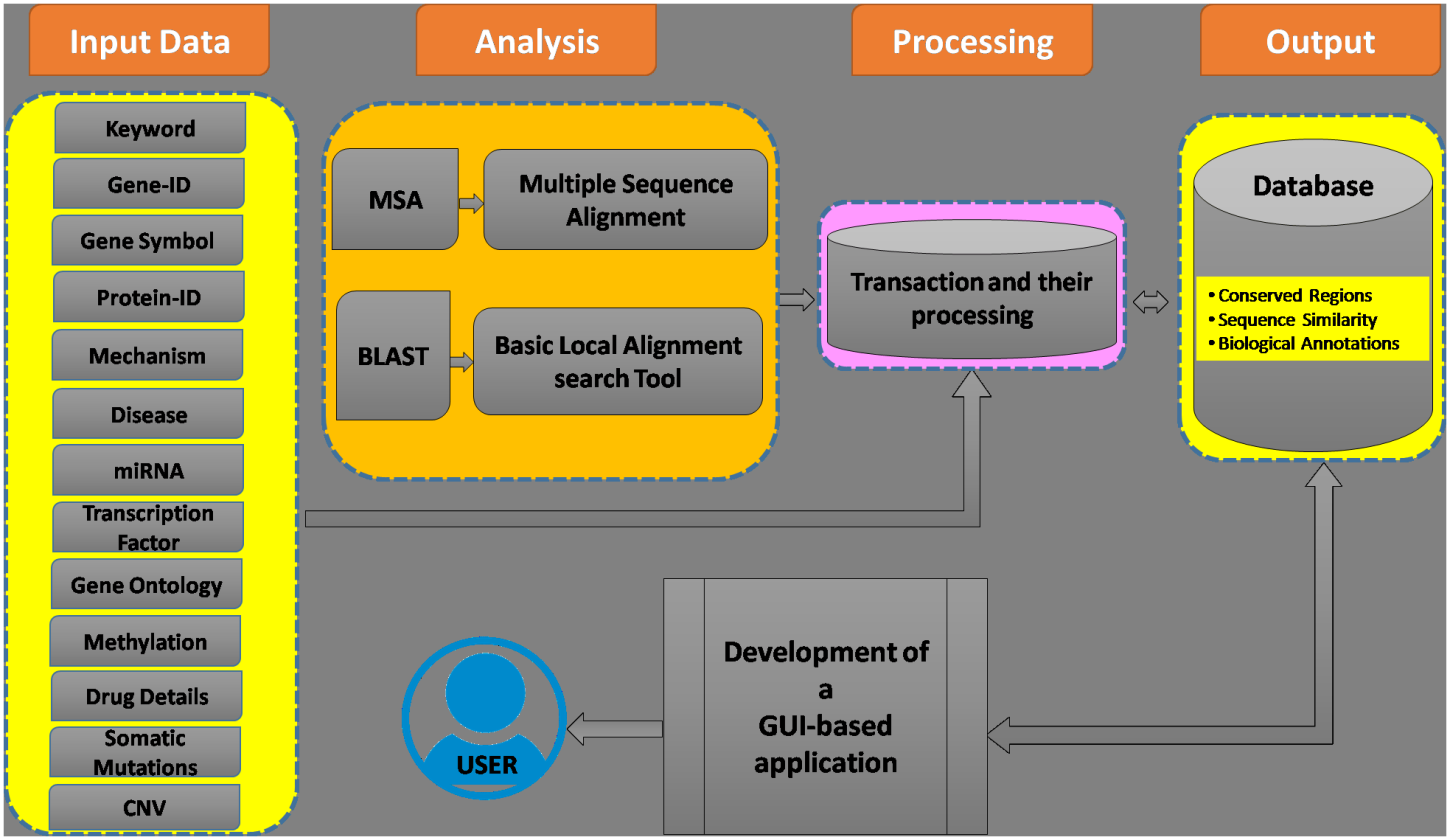
Search filters to retrieve the data. Various search option that includes search by GENE-ID, GENE SYMBOL, PROTEIN-ID, MECHANISM, DISEASE, TRANSCRIPTION FACTOR, GENE ONTOLOGIES, MIRNA, METHYLATION, DRUG DETAILS, COPY NUMBER VARIATION, SOMATIC MUTATIONS.

DREMECELS is a collection of 156 DNA repair (MMR and BER) genes associated with diseases. It provides an easy and effective method to search and retrieve data of associated genes along with other information as shown in Fig 2. The data in DREMECELS can be retrieved using the query search option in 12 different ways: (1) search by gene ID, which can be queried using the standard GenBank ID; (2) search by the gene symbol, which can be queried using the standard gene symbol; (3) search by protein ID, which can be queried using the UniProt accession ID; (4) search by the mechanism, preferably using BER, MMR, or both BER/MMR mechanisms; (5) search by disease, specifically for CRC, EC, LS, and other associated diseases; (6) search by gene ontology, which can be queried using GO ID; (7) search by miRNA, which can be queried using the miRNA symbol; (8) search by the transcription factor, which can be queried using the standard HGNC-approved symbol; (9) search by methylation, which can be queried for methylated, hypo and hypermethylated genes; (10) search by drug details, which can be queried using the drug name; (11) search by CNV, which can be queried either by disease name or by gene name; (12) search by somatic mutations, which can be queried using six mutations types which includes substitution-missense, substitution-intronic, substitution-nonsense, substitution-coding silent, deletion-frameshift, and deletion-in frame.

After query submission, if the search query matches the entry in DREMECELS, users will be redirected to the results page. The results page displays majorly three outputs: (1) comprehensive information regarding genes, which includes gene ID, gene symbol, protein ID, protein name, mechanism, annotation, transcription factor, pathways, disease, PubMed ID, miRNA, interaction ID, PDB ID, ENSEMBL ID, OMIM ID, conserved domains, and external link to the gene card; (2) pictorial representation of the interacting genes through STRING [28] embedded in the portal, and (3) MSA results obtained through MUSCLE. However, methylation and drug data are provided on a separate result page where the table includes information regarding the gene ID, gene symbol, chromosome number (start and end positions), methylation percentage (%), experimental method used to locate the regions of methylation, disease type that methylation is associated with, reference ID, and CpG number. Drug details include drug ID that is manually created, drug name, tissue at which the drug is targeted, disease type for which the drug is implicated, cell line, IC-50 value (that measures the effectiveness of a substance in inhibiting a specific biological or biochemical function), molecular targets, PubChem ID, and data source. CNV details include sample, gene, expression (classified as under, over or normal), CN_Type (either loss or gain), copy number (total copy number for the specified CNV (major allele + minor allele counts) and copy number segment position which displays genomic position of the CNV. Somatic mutations details include gene, transcript, sample name, amino acid mutation (change that has occurred in the peptide sequence due to mutation), somatic mutations which include six major type of mutations (substitution-missense (mutation resulting in an alternate codon), substitution-intronic (mutation outside the coding domains), substitution-nonsense (mutation resulting in termination codon), substitution-coding silent (mutation which encodes the same amino acid as the wild type codon) deletion-frameshift (deletion of nucleotides which alters the translation frame, changes downstream peptide sequence resulting in premature termination) and deletion-in frame (deletion of nucleotides which doesn’t affect the gene’s translation frame, leaving the downstream peptide sequence intact)), and disease. Furthermore, a customized BLAST [29] tool is integrated in the database, particularly for performing protein BLAST; this tool searches a user-defined protein query against sequences available in the protein database. It may be useful in characterizing hypothetical sequences and acquiring homologous sequences from the database on the basis of sequence similarity. Standard parameters for protein BLAST have been incorporated to make the search smooth and effective.

DREMECELS is being planned and developed keeping in mind the requirements of researchers working in the area of three specific diseases mentioned above. Unique part of it is the manually curated information where unessential details were removed to provide specific and useful information to the researchers. Additionally all the important parameters and features have been linked to their primary or original resources to connect users to the extensive pool of information, which could have not been compiled in this repository. Download option has also been provided for the users to download all kind of data available through various options (http://bioinfoindia.org/dremecels/download.php). Also the information content for all three diseases is a very special and unique feature of this database. These unique features make DREMECELS a value aided resource for the researchers and biomedical scientists working on DNA repair systems for CRC, EC, and LS.

## 4. Conclusion

The DREMECELS is the first endeavor to build a database particularly dedicated to colorectal and endometrial cancers, which is associated with Lynch syndrome. We compiled all manually curated data regarding these malignancies for providing a knowledge-based resource that will allow researchers and clinicians to have a biological overview of the genes involved in these diseases. This database would save time and efforts of researchers involved in the field, and thus will facilitate in biological discoveries. The database will be updated regularly to provide state-of-the-art information to the academicians and researchers. Currently, database contains 156 unique genes that are supported by literature references. We have tried our best to provide the maximum possible information in the database and to represent each record in a systematic and organized form so that it provides clarity, ease of access, download and fast browsing capability.
